# New Frontiers in Materials Design for Laser Additive Manufacturing

**DOI:** 10.3390/ma15176172

**Published:** 2022-09-05

**Authors:** Silja-Katharina Rittinghaus, Eric A. Jägle, Manfred Schmid, Bilal Gökce

**Affiliations:** 1 Chair of Materials Science and Additive Manufacturing, School of Mechanical Engineering and Safety Engineering, University of Wuppertal, 42119 Wuppertal, Germany; 2 Institute of Materials Science, Universität der Bundeswehr München, 85579 Neubiberg, Germany; 3 Innovation Center for Additive Manufacturing, Inspire AG, 9014 St. Gallen, Switzerland

Laser-based additive manufacturing (LAM) in all its variations is now being established as a technique for manufacturing components from various material types and alloys. However, the materials regularly used in these processes were developed for conventional manufacturing processes (e.g., casting, injection molding, thermal spraying). They are therefore not optimized for the characteristic process environments in laser additive manufacturing, so some of the great potentials of this manufacturing technology remain untapped. The urgent need for new materials throughout the industry is reflected in current trends and market studies, e.g., [1], as well as intensified research activities on material development for (L)AM.

The task of designing new materials is very challenging and, to be truly successful, requires interdisciplinary collaboration between experts from a wide range of disciplines (cf. Figure 1). Promising approaches to research include additivation and modification of existing commercial base materials, typically powders, but also creating completely new alloys starting with phase modeling and basic chemical reactions. Special properties to be taken into account are, for example, powder material properties, flowability properties, melt pool and flow characteristics, as well as solidification conditions. The consideration of industrial requirements such as high efficiency, reproducibility, and precision is highly essential for a holistic, sustainable approach.

With regard to metallic materials, one long-lasting challenge is to modify previously un- or hardly processable materials in such a way that their defect-free consolidation becomes possible, e.g., by adding zirconia or boride nanoparticles to highly crack-susceptible alloys such as Al7075 and Al606, as it is successfully being pursued by [5], and thus to expand the application range of LAM. This goes hand in hand with research and development on the processes themselves, as, e.g., proposed by [6], accepting the challenge of manufacturing Mg-alloys with laser-based powder bed fusion (LPBF). Further developments and innovations in the fields of systems engineering and photonics support these efforts, such as the use of ultrasound [7] or green laser radiation [8]. Similarly, many studies are currently in progress to determine how the special temperature conditions in the process (cyclic heating, rapid cooling) can be used in a targeted manner to produce special microstructures, for example, precipitates or extremely fine structures, like in Al-Ni eutectic alloys investigated by [9] or even to adjust properties locally in the component, as can be derived from results of [10] on Al-Fe. Intrinsic heat effects can be used to trigger material transformations if they are cleverly controlled [11] and thus enable new component designs. Likewise, a targeted scan strategy and process parameter control allows us to locally promote and select crystallographic orientations and phase compositions (e.g., [12,13,14]).

Due to the increasing computational capacity and performance, numerical methods are increasingly used to represent and predict material behavior. Modeling of non-equilibrium states, such as those encountered in LAM, is a valuable complement to classical phase diagrams and paves the way for digital material development. Additivation, primarily with nano-sized additional particles that are not dissolved in the melt pool and act as nuclei and/or reinforcement in the resulting microstructure, is another growing field of research for LAM of metals. Oxide dispersion strengthening (ODS) is an evolving example of how material development benefits from AM by providing a method to produce these types of composites more economically or at all. These advances also benefit from sophisticated modeling, e.g., [2] propose a model to predict the nanoparticle’s exact location in solidified LAM material. Another promising approach to modify powders at the nano level is to systematically coat them to improve process behavior, like, e.g., investigated by [3] on maraging tool steel.

Nanoparticles are also quite exciting for the AM of polymer materials [15]. A lot of research focuses on semi-crystalline polyamide 12 (PA12). The authors of [16] were able to prove in their investigations that even small amounts of added carbon nanoparticles can significantly influence the mechanical properties without adversely changing the crystallization behavior. Metallic or ceramic additives in micro- or nanoscale embedded in a polymer matrix can be used to produce highly specialized parts even with new functionalities, like, e.g., magnetic characteristics [17]. Achieving the desired distribution, however, is still challenging in many applications. Polylactide acid (PLA) is another polymer with increasing popularity in AM. Known to be both biocompatible and biodegradable, the scope in [18] to also make the manufacturing route more environmentally friendly is both obvious and ambitious.

In both metal and plastic AM, sustainability is playing an increasingly important role in material selection and development. Efforts in the metals sector often include attempts to substitute elements that are environmentally and ethically critical or that have an insecure supply chain, such as rare earths, and to improve recycling routes for materials along the entire process chain and components. With regard to polymers, research into the more ecological production of raw materials is coming to the fore, as well as investigations into materials that are biodegradable or easier to recycle or reuse.

Another material class-independent trend is the increasing use of modeling to map process-dependent material behavior, thus reducing experimental effort and shortening development cycles. For this, many correlations remain to be fully understood, making fundamental work like, e.g., [19], which focuses on capillary phenomena, essential. This is also accompanied by ever-increasing demands on measurement technology, which motivates work like, e.g., those of [20], which contributes to learning about fast phase transition kinetics by using advanced measurement techniques. In general, in situ techniques for observing microstructure development during the process to gain a deeper knowledge of process–property correlations are on the rise and quite successful [21].

As in any field of research, a collaboration between different researchers is the key to innovation. Increasing digitization has advanced the rapid availability of data worldwide and is accelerating developments on a massive scale. To assure consistency and validate developed methods, interlaboratory studies like the one proposed by [4] are useful evaluation tools that benefit from a high number of researchers and laboratories involved. Additionally, it becomes increasingly crucial to keep track of the growing volumes of data and make the best possible use of them, research data management is also gaining more and more importance.

## Figures and Tables

**Figure 1:**
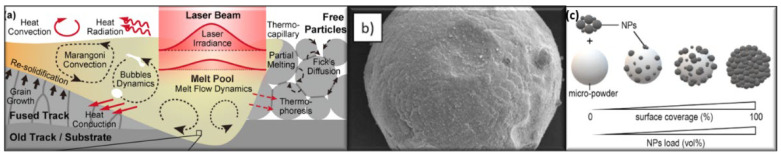
(**a**) Schematic of interactive physical phenomena during a stable LPBF process [2]. (**b**) scanning electron microscopy (SEM) picture of 1.2709 at 10,000× *g* magnification coated with 1 vol.% SiC [3]. (**c**) Illustration of the surface coverage by micro powders with increasing nanoparticle loading (vol%) [4].
